# Under the sun: adaptation effects to changes in facial complexion

**DOI:** 10.1186/s40359-023-01148-9

**Published:** 2023-04-01

**Authors:** Sandra Utz, Ronja Mueller, Tilo Strobach, Claus-Christian Carbon

**Affiliations:** 1 Department of General Psychology and Methodology, University of Bamberg, Markusplatz 3, 96047 Bamberg, Germany; 2 Bamberg Graduate School of Affective and Cognitive Sciences (BaGrACS), University of Bamberg, Bamberg, Germany; 3 Research Group EPÆG (Ergonomics, Psychological Æsthetics, Gestalt), Bamberg, Germany; 4 Department of Psychology/Institute for Cognitive and Affective Neuroscience (ICAN), Medical School Hamburg, Hamburg, Germany

**Keywords:** Complexion, Face adaptation, Face perception, Face memory, Face recognition, Skin color

## Abstract

**Background:**

Many Western people enjoy sunshine, and through the sun’s stimulated increase in melanin, the skin tone or skin complexion will darken (and lighten again during winter). Although the initial salience of such a new look is remarkable – especially in the face – we seem to adapt to this new look relatively quickly. Research on face adaptation in general repeatedly showed that the inspection of manipulated versions of faces (so-called adaptor faces) leads to a change of the perception of subsequently presented faces. The present study investigates face adaptation to very natural changes in faces such as changes in complexion.

**Methods:**

During the adaptation phase in the present study, participants saw faces with either strongly increased or decreased complexion. After a pause of 5 min, participants had to identify the veridical (non-manipulated) face out of two faces (a face slightly manipulated in complexion combined with the non-manipulated face) during a test phase.

**Results:**

Results show strong adaptation effects to decreased complexion intensities.

**Discussion:**

It seems that we are updating our facial representations in memory quite quickly (i.e., optimizing our processing through adaptation) and seem to sustain those new representations over a certain timespan (at least 5 min). Our results demonstrate that changes in complexion draw our attention for deeper analysis (at least with decreased complexion). However, it loses its informative quality quickly via fast and relatively sustainable adaptation.

## Introduction

Being exposed to the summer sunshine in Hollywood considerably changes the appearance of the faces of some well-known celebrities. For some of them, freckles start to spread all over the faces, but the more general change is the darkening of the skin tone in contrast to the light complexion during winter. What happens is that once the skin is exposed to UV radiation, it increases melanin production. This increase in melanin can cause the complexion to darken to protect the skin from further damage [1]. After some exposure, people surprisingly quickly adapt to those new facial features in otherwise quite familiar faces. The same face with the light winter complexion would now be perceived as almost “unnatural” and other tanned faces as completely “natural”.

This adaptation effect makes complexion an interesting case of dynamically changing face properties (through exposure to sunshine), along with freckles and other more linear changes like aging effects (wrinkles, skin quality, and macules). A darker complexion is often a desirable aim in Western societies (artificially utilizing a sunray lamp as well as naturally by the sun). Driving this desire might be the idea that a tanned complexion makes people appear attractive (see, e.g. [2],) or more prestigious as they can afford time and money for tanning shops (and holidays in sunny regions of the world). However, tanning seems to be a dead issue since it is actually not a lasting effect (as anecdotally reported above): after a brief period, adaptation processes to complexion seem to be taking place, and others will not even perceive the effect anymore. Such adaptation is known to be a mechanism for optimizing the processing of our actual environment. Therefore, it makes sense to adapt to changes such as changes in complexion quite quickly. Of course, it is evident that the change of complexion can be a whole-body phenomenon. However, the present study focuses on a body part, where this change will become obvious within the first seconds of an encounter since almost everyone (with few exemptions) is looking firstly at a person’s face.

Webster and MacLin [3] were the first to investigate our ability to adapt to manipulations in faces (so-called face adaptation effects) and together with a considerable amount of follow-up studies (e.g., [4–7]), it was repeatedly shown that thoroughly inspecting manipulated faces (so-called adaptor faces) leads to changes of the perception of subsequently presented faces. As a result of this adaptation process, original face images are perceived as manipulated in the opposite direction of the adaptor, while images more similar to the adaptor are perceived as normal.

Bao and Engel [8] were the first to show that different temporally-tuned processes are responsible for how the visual system adapts to dynamic changes in the environment such as changes in faces. Mesik, Bao, and Engel [9] investigated this timescale of visual adaptation in more detail. According to the authors, adaptation seems to result from a combination of multiple temporally-tuned processes. Some processes arise earlier in the processing course and are more short-lived in nature and some processes arise later and are more sustainable. Following this theory, sensitivity is proportional to the sum of outputs of the multiple components. For example, long adaptation to high contrast causes short- and long-lasting components to signal a sensitivity decrease. If participants then adapt to low contrast (so-called de-adaptation), the short-term components signal a sensitivity increase, stopping the effects of the more long-lasting components. Afterward, the short-term mechanisms will return to baseline, leading to a recovery of the long-term adaptation effects without the participant being aware of this process [10]. Therefore, the finding of this spontaneous recovery argues for a multiple mechanism theory (e.g., [8, 9]). This idea of different temporally-tuned processes in the context of adaptation was also confirmed for more natural facial categories (such as emotional expressions, eye gaze, facial gender, and facial identity, [11]).

The long-lasting processes in the “temporal-tuning theory” suggest that adaptation is not just based on transient sensory processes but on longer-term alterations of the memory. Strong support for this assumption also comes from studies investigating the robustness of adaptation effects across several days. Carbon and Ditye [12], for example, were able to detect adaptation effects on configurally distorted celebrity faces when implementing a delay of one week between adaptation and test phases. Since the robustness and sustainability of those effects were quite surprising, the present study also focuses on the memory component of facial adaptation effects and not on the more perceptual component of these effects. This also aligns with the most recent Episodic Prototype Model (EPM) by Schneider and Carbon [13], proposing that our mental representations of faces are more flexible than expected. The proposed episodic facial prototypes consist of only a few needed mental representations leading to efficient storage and more flexibility with dynamic changes in faces (such as tanning), i.e., prototypes can be changed or adapted more easily (and quickly) than previously assumed. Essentially, these effects are attributed to memory-based effects in order to assist the reliable recognition in later episodes where we can rely on the most updated version of an outward facial appearance.

Moreover, the face adaptation effect can even be transferred to faces not previously seen during adaptation, e.g., different facial depictions of the same identity or even different identities (shown by, e.g., [7, 12, 14–17]). Based on these transfer effects, it is highly likely that adaptation seems not to be based on a pictorial but rather on a higher and more abstract or structural level. Also, since effects are robust over time and transferable across different depictions and identities, adaptation seems not to be caused by simple aftereffects on a retinal level or by simple episodic memory processes (e.g., recency effects), but more likely by higher cognitive processes responsible for the permanent update of our mental memory representations. Rather, facial adaptation can be explained in the following way: If a strongly manipulated familiar face is inspected, the internal representation of this face is immediately and automatically updated by integrating the newly presented exemplar. Suppose the original face out of several presented faces has to be detected afterward. In that case, a presented face being more similar to the adaptor face is then perceived as more veridical (i.e., representing the original face) than the original face itself. The original face will be perceived as slightly manipulated in the opposite direction to the adaptor face [18] since it does not correspond to the (updated) representation anymore.

Adaptation could be shown not only to manipulations of configural information in faces (see review [18, 19]), but also to combined manipulations of configural and non-configural (i.e., featural information; e.g. [20],). The studies by Mueller et al. [21, 22] are the first studies that systematically show that manipulating non-configural face information (faces were manipulated regarding brightness or saturation, respectively) leads to relatively robust (over time) and transferable adaptation effects. Effects found by Mueller et al. [21, 22] were specific for upright faces, in contrast to scrambled and inverted faces. Non-configural face information, therefore, show adaptation effects and seems to play an important role in the storage of faces.

The present study further examined adaptation effects, which can be attributed to the area of non-configural face adaptation. Our main research aim was to investigate relevant changes in faces actually happening in our everyday life, in contrast to Mueller et al. [21, 22] using more artificial brightness (a change in perceived intensity of a visual stimulus; e.g. [23],) or saturation manipulations in faces. To realize changes happening in everyday life, we used ecologically valid material to test adaptation effects regarding changes in complexion (i.e., the color or appearance of a person’s skin). Although complexion can be a phenomenon regarding the whole body, only faces were used in the present study since faces are immediately inspected when we meet someone (and if most of the rest of the body is covered by clothes). We either lightened or darkened the skin tone of the respective faces to simulate natural changes in complexion realistically. Well-known celebrities were used as familiar faces because participants were already equipped with an established representation of these faces. During adaptation, participants were familiarized with the faces with either strongly lightened complexion, strongly darkened complexion, or non-manipulated face versions. Following adaptation to strongly manipulated faces, we expect an alteration of the participant’s representation of the respective faces towards the adaptor. Thus, the original face would appear in a manipulated way in a direction opposite to the adaptor (e.g., the original face would seem to be increased in complexion after adaptation to a face with a decreased complexion, and vice versa). As a consequence, during a test phase, we expect participants to select a face version more similar to the adaptor (e.g., with a slightly decreased complexion) to be the veridical face when presented with the two options (original and slightly manipulated face version).

Robustness, as well as transferability of these adaptation effects, were also investigated. By using a paradigm separating adaptation and test phase by approximately five minutes (see [14]), we ensured that any effects were not merely based on ultra-short-term perceptual effects. Since we were also interested in finding out whether possible effects are based more on pictorial (same faces presented in adaptation and test phase) versus structural effects, we additionally employed different depictions of the same celebrity (structural transfer) or completely different identities (cross-identity transfer) in the adaptation and test phases. These transfer conditions can clarify whether adaptation effects are either image- or identity-specific. With these transfer conditions, we can even address higher and more abstract face concepts (e.g., a generic norm of faces). Robustness and transferability of adaptation effects to dynamically changing facial information help us to better understand how non-configural face information might be represented in memory. In line with Mueller et al. [21], we expect transfer effects, at least on the pictorial level, with comparable robustness over time.

## Materials and method

### Participants

Thirty-six participants (31 female, five male) were tested with ages ranging from 18–57 years (*M*_*age*_ = 24.6 years; *SD*_*age*_ = 7.0; the sample was mainly around 24 years of age, excluding the 57-year old participant substantially decreased the age range: *M*_*age*_ = 23.7 years; *SD*_*age*_ = 4.4; range: 18–41 years; still, we included all participants from the sample as we did not observe any oddity in the data and to achieve a fully balanced experimental design). This number of participants was calculated a priori via power analysis [24] based on a mixed-design analysis of variance (ANOVA) with a 3 (between-subjects) × 3 (within-subjects) factor design being able to detect a medium effect size *f* of 0.25 [25] given an α = 0.05 and a test power (1 − β) = 0.80. Similarly sized effects for the factor *adaptation condition* (the main focus in our study) was revealed in previous studies (e.g., [17]: ${\upeta }_{\mathrm{p}}^{2}$= 0.316—medium effect; [12]: ${\upeta }_{\mathrm{p}}^{2}$= 0.350—medium-to-large effect). All participants had normal or corrected-to-normal vision (assessed by a standard Snellen eye chart test) and normal color vision (assessed by a short version of the Ishihara color test). All participants were White Europeans, all of them being undergraduates of the University of Bamberg. They received course credit points for their participation or were alternatively paid 15 €. They had no prior experience with the present task and were naïve to the purpose of this experiment.

### Apparatus

Participants were seated approximately 60 cm in front of a 61.2 cm EIZO ColorEdgeCG245W-LC monitor running at a screen resolution of 1,920 × 1,200 pixels with a refresh rate of 60 Hz controlled by a standard PC. Participants responded to the experimental task by pushing keyboard buttons (letters). Stimuli, trials, and experimental blocks were created with the up-to-date version of PsychoPy (v3.0 [26];), ensuring high precision in executing the correct timing of the study.

### Stimuli

**Fig. 1:**
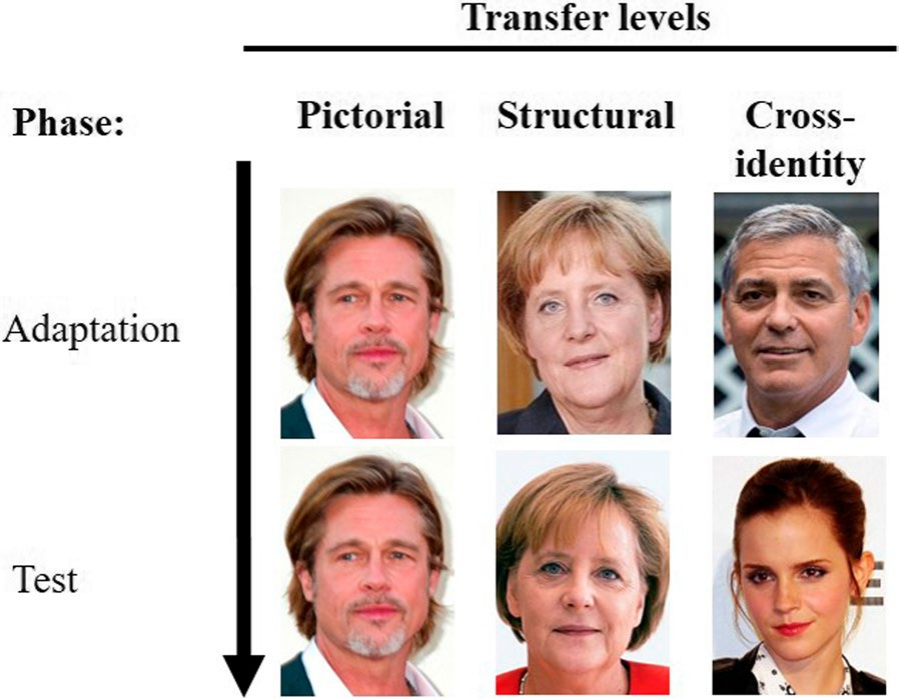
Examples for the three transfer levels (pictorial, structure, & cross-identity), similar to Mueller et al. [21, 22] (images are just examples and not used within the study; they are taken from https://commons.wikimedia.org under CC BY-SA 3.0 license)

A pre-study was conducted to select celebrities for our target participant group (undergraduate students). For this pre-study, seventy facial depictions of (potential) celebrities were chosen according to the following criteria: full face in high resolution, color (not black-and-white), frontal view, straight gaze, no facial features covered with hair, and no glasses. Celebrities were then presented to 92 participants^1^ (66 female, 26 male; *M*_age_ = 21.7, range: 18–30 years) in random order and participants had to write down the name of the depicted celebrity and judge their familiarity on a 5-point Likert scale (1 = *unfamiliar* to 5 = *very familiar*). The 30 celebrities^2^ with the highest familiarity were then chosen as stimulus material for the main study and randomly divided into three sets of faces (I, II; III). Furthermore, two depictions (A, B) of the same person were selected, resulting in six different stimulus sets. The two depictions showed a full face in frontal view, a straight gaze, no glasses, and no hair covering any facial features. However, both images were taken on different occasions. This way, we ensured that both images differed significantly in their depiction of the same identity. This was necessary to realize the within-subjects factor of the different transfer levels. The three different transfer levels varied in the overlapping information between images presented in the adaptation and test phase (Fig. 1). On the pictorial transfer level, stimuli in the adaptation and test phase are pictorially identical (e.g., presenting picture A of Brad Pitt as adaptor and test stimulus). On the structural transfer level, the same identity is used in the adaptation and the test phase, but employing a different depiction (e.g., presenting picture A of Angela Merkel as an adaptor and picture B of Angela Merkel as test stimulus). On the cross-identity transfer level, different identities are presented in the adaptation and the test phase (e.g., presenting picture A of George Clooney as an adaptor and presenting picture A of Emma Watson as test stimulus). These photographs were presented at a size of approximately 210 × 288 pixels^3^ in all experimental phases.

**Fig. 2:**
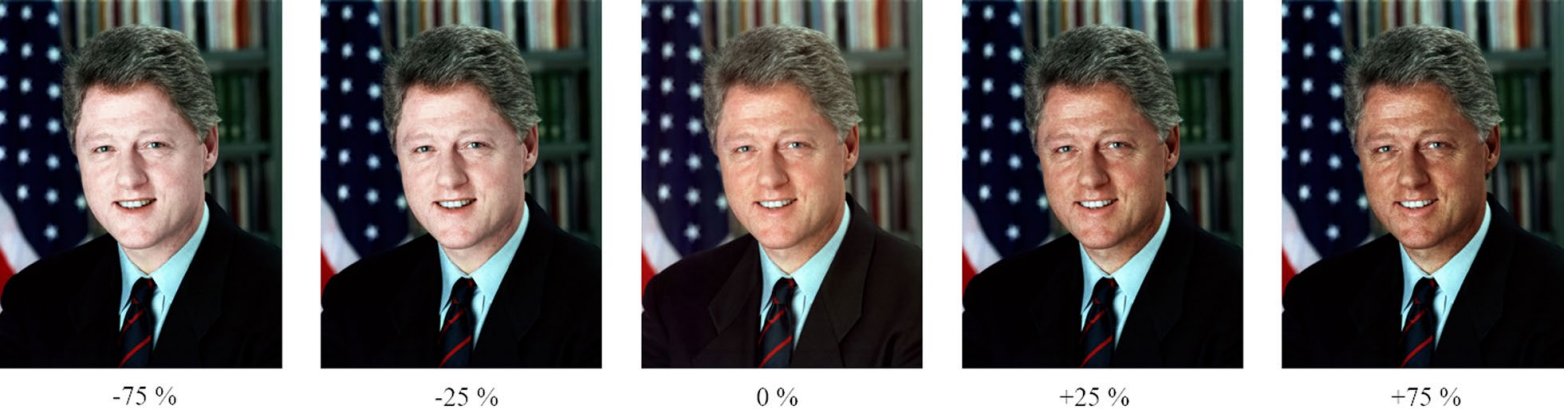
Example for the different complexion intensities ranging from -75% to + 75%. The non-manipulated image (0%) was used as the original picture (adaptation, test phase), extreme intensities (-75%, + 75%) were used for the adaptation phase, and middle intensities (-25%, + 25%) in combination with the original picture (0%) for the test phase. (Note that these images were not used in the original study but are displayed here for illustrative purposes only; the base image is taken from https://commons.wikimedia.org under CC BY-SA 3.0 license—accordingly, our transformed versions will also be available via CC BY-SA 3.0 ShareAlike license)

The intensity of complexion was manipulated using Adobe Photoshop CC (Version 19.0), resulting in five different versions from fair to dark complexion (examples see Fig. 2). Note also that the original picture was always the basis of the complexion manipulation. The actual complexion of the adaptor is, therefore, always dependent on the complexion of the original picture, i.e., the value of the change from the original picture to the manipulated picture is held constant across manipulations.

*Adjustment of base stimuli.* As different lights and contrast can vary drastically for each base image and thus could influence the goal for a standardized image perception negatively due to color differences in our results of skin tones, we tried to minimize differences in the color spectrum for each image. This was the reason that we used a color palette picked from one of our specific base images (we chose one picture of Angela Merkel as the baseline) and applied this color spectrum on all the other stimuli to reduce differences in color spectrum, which overall resulted in more balanced blue tones and slightly increased red tones in each image. To standardize the images, we adjusted color tones. Therefore, we first reduced the baseline image to a primary color image version that only consisted of its three primary tones, i.e., dark, mid-, and light color tones (in the case of our chosen baseline image, the tones were identified were, respectively, as the following: dark color tone: #2d231f – B (brightness) 18%, mid color tone: #a18574 – B 63%, light color tone: #d4c6bd – B 83%). We then applied this color tone spectrum to all of the base images by using a gradient map.

*Generation of image variants with different complexion intensities.* Different complexion intensities variants were generated by employing the *Adobe Photoshops Camera Raw Filter* where we set the same camera raw adjustments for each image for the extreme light skin tones by manipulating the parametric gradation curves regarding light tones (-35) and depts (+ 45) and HSL/Grey scale parameters (H: -15, S: -40, L: + 100), applied to the skin areas. We generated the extreme dark skin tones variants through three different adjustment layers applied to the skin areas, i.e., Black/White (Reds: -60, Yellows: + 60, Greens: + 40, Cyan: + 60, Blue: + 20, Magenta: + 80), Hue/Saturation (Color: 0, Saturation: 30, Brightness: -75), and the Gradient Map (Color Code: #3b1e07 from position 0% to100%). We carefully ascertained that the no-skin areas were not affected by all these adjustments using layer masks. Middle intensities variants were generated by setting the Hue/Saturation brightness parameter to -25. See Fig. 2 for an illustration of the results of these manipulations.

**Fig. 3:**
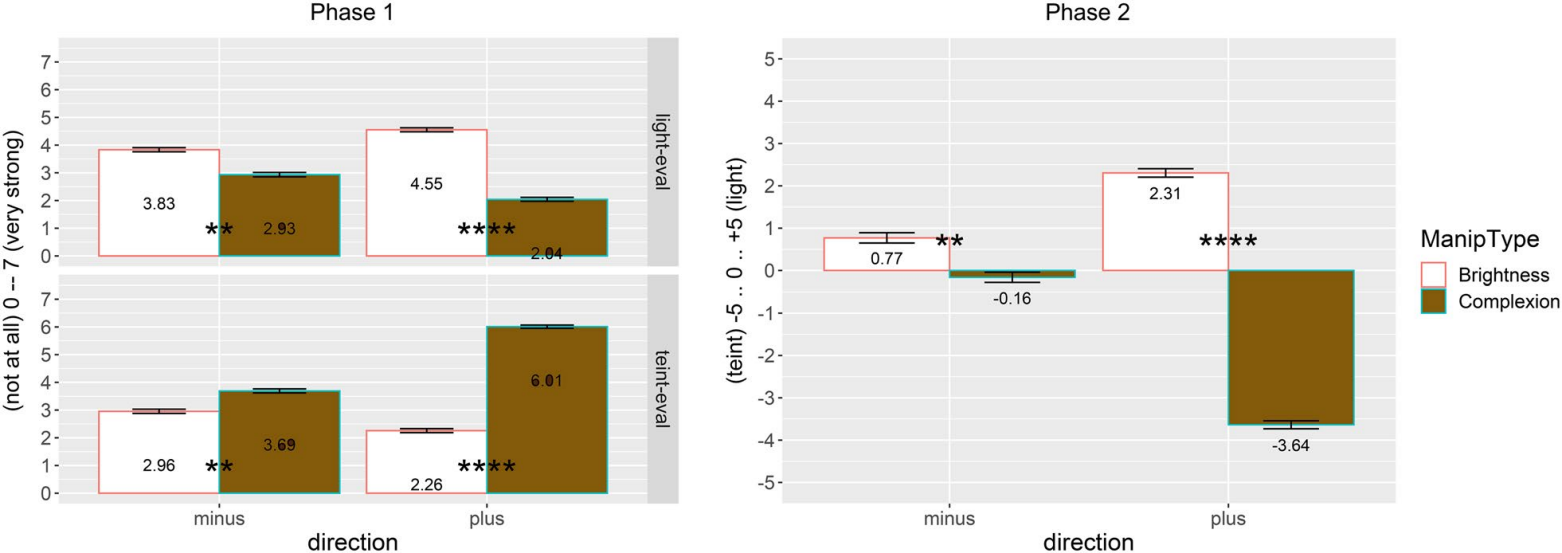
Results of the two experimental phases testing the actually perceived differences between brightness and complexion manipulated stimuli. Asterisks indicate statistical significance based on *t*-tests, **** *p* < 0.0001, ** *p* < 0.01

To ensure that face manipulations were actually perceived as changes in complexion and not as changes in brightness, forty-two participants rated all stimuli that were presented in the adaptation phase of the study by Mueller et al. [21] and of the present study within two experimental phases. Within the first phase, two faces were presented (one original & one manipulated; 660 px high at 96 dpi) at the same time (distance 100 px). Participants had to indicate if the manipulated picture was the result of a change in complexion or in brightness (0 = *not at all* to 7 = *very strong*). In the second phase, only one picture was presented and participants had to indicate how likely this picture was changed with regard to complexion (-5) to brightness (+ 5). Results (depicted in Fig. 3) clearly show that participants actually perceive a difference between the two manipulations and can clearly identify if the brightness or the complexion of the picture was manipulated.

### Procedure

**Fig. 4:**
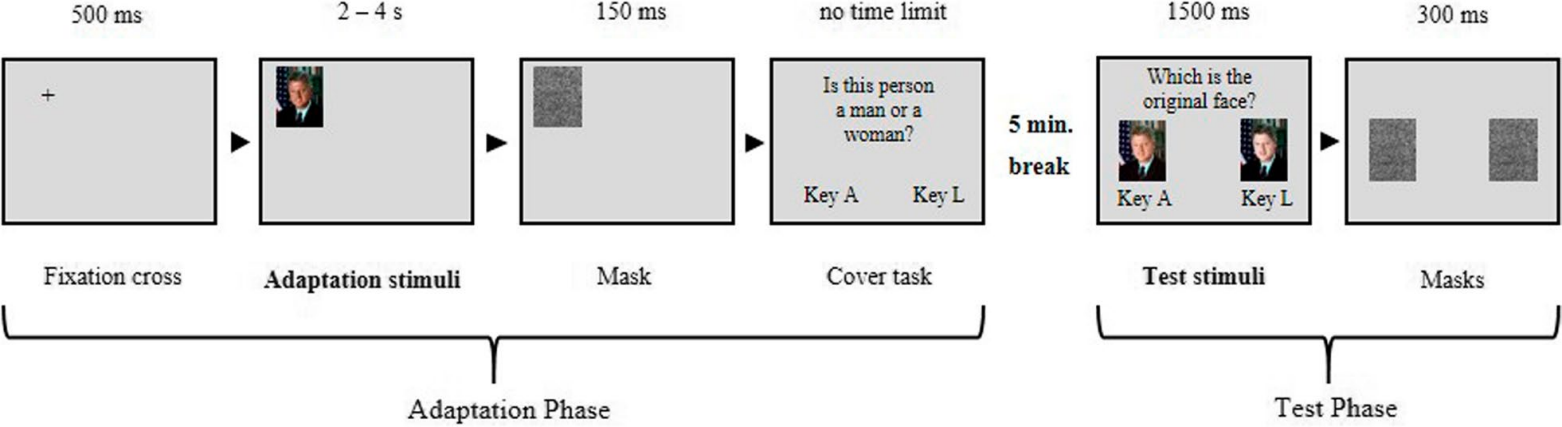
Schematic depiction of the trial procedure for the adaptation and test phase

The study consisted of four parts: an adaptation phase, a 5-min reading break, a test phase, and a familiarity rating task (for the whole procedure see Fig. 4).

*Adaptation phase*. Faces were presented as adaptors in three versions: either original (ORIGINAL), with strongly darkened complexion (+ 75%; PLUS EXTREME), or strongly lightened complexion (-75%; MINUS EXTREME). Participants were randomly assigned to one of the adaptation conditions (between-subjects factor). To control for retinal effects, faces were presented at six different monitor positions (top-left, top-center, top-right, bottom-left, bottom-center, and bottom-right). Before each trial of the adaptation phase, a fixation cross (displayed for 500 ms and placed in the center of the subsequent stimulus position) was displayed before presenting the adaptor. To increase inspection times while avoiding fatigue effects, the presentation time of adaptors was varied (2, 3, or 4 s; adapted from [14]). Masks appeared afterward for 150 ms. Positions and inspection times were randomized throughout the experiment. Each adaptor was presented 18 times (3 presentation times × 6 positions). Each participant adapted to two sets of faces, i.e., pictures A or B of two (out of the three) sets of faces (I, II, III). One set of faces was not shown during adaptation. Participants’ task was to decide whether the depicted person was male (by pressing the key “A”) or female (by pressing the key “L”). A total of 360 trials was presented (20 faces × 6 positions × 3 presentation times). After half of the trials (i.e., 180 trials), participants were allowed to take a break and were free to decide when they wanted to start the second half of the adaptation phase.

*5-min break*. During the break in-between adaptation and test phase, the participants’ task was to read a (to the current experiment) completely unrelated text about the geography of the Republic of Kenya (source: https://www.lernhelfer.de/schuelerlexikon/geografie/artikel/republik-kenia#). The reading task aimed to prevent the mental recall of the previously seen faces. After five minutes of reading, participants were interrupted by the experimenter and were asked to enter the test phase.

*Test phase*. After the instruction, participants could start the test phase by pressing the space bar on the keyboard. In a two-alternative-forced-choice (2-AFC) task, two different versions of the same picture were presented. The original picture (ORIGINAL) was always accompanied by either a picture with slightly darkened complexion (+ 25%; PLUS) or slightly lightened complexion (− 25%; MINUS) on two spatial positions (position left: 720 × 960 pixels, position right: 1200 × 960 pixels) with the spatial position counterbalanced over the whole task. Independently of the adaptation group, all participants were confronted with MINUS as well as PLUS images. Pictures were presented for 1,500 ms, and masks appeared afterward for 300 ms. Participants’ task was then to decide if the left or the right of the presented pictures represents the original picture by pressing “A” or “L”, respectively. Participants were explicitly instructed to base their decision on their memory about the celebrity (e.g., images seen in the media). During the test phase always three sets of pictures were presented (two sets participants adapted to, and one set participants did not adapt to), resulting in a total of 30 faces. To apply the three different transfer levels, either the same or the corresponding pictures A or B of each set (I, II, III) were presented depending on the image sets during adaptation. As mentioned above, for the pictorial transfer level, the same picture version was presented as in the adaptation phase, for the structural transfer level, the other picture version was presented, and for the cross-identity transfer level, images of the set not shown during adaptation were used as test targets. In total, 120 trials (30 faces × 4 spatial arrangements) were presented.

*Familiarity rating task*. Participants had to judge the celebrities according to their familiarity (“Are you familiar with this celebrity from the media?”). Participants responded with either “yes” (key “A”) or “no” (key “L”). The aim of this task was to ensure that the participants based their selection about the veridicality of the identities in the experiment on an internal (stable) mental representation.

The entire study, including the adaptation phase, break, test phase, familiarity rating task, plus the debriefing at the end of the study lasted about 1.5 h in total.

## Results

**Fig. 5:**
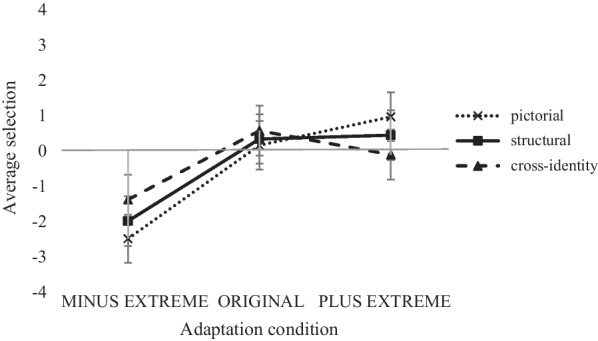
Illustration of the average selection (mean complexion intensity in % of the selected test stimulus) for the three adaptation conditions (MINUS EXTREME, ORIGINAL, PLUS EXTREME) and the three transfer levels (pictorial, structural, cross-identity). Error bars represent ± 1 standard error of the mean

In the test phase, trials with reaction times (RTs) faster than 200 ms and slower than 3 *SD*s above the individual RT mean were excluded from further analyses (resulting in an average data loss of 2.0%; *SD* = 3.0). On average, 95.0% of the celebrity faces were considered to be familiar (familiarity rating task). Trials displaying unfamiliar celebrities to participants were excluded from further analyses, since our memory probably contains only stable mental representations of familiar identities. Target selection was the dependent variable. Target selection was scored according to the version of the selected face (complexion intensity: MINUS = -25%; ORIGINAL = 0%; PLUS =  + 25%). That is, if a participant selected the MINUS face out of the two faces presented in the current trial, this trial received the score “-25”, if a participant selected the ORIGINAL face out of the two faces presented in the current trial, this trial received the score “0”, and if a participant selected the PLUS face out of the two faces presented in the current trial, this trial received the score “ + 25”. Average selection represented the mean complexion intensity of the selected test stimulus in % (note that the maximum was -12.5% and + 12.5%). A mixed-design ANOVA with the within-subjects factor *transfer level* (pictorial, structural, cross-identity) and the between-subjects factor *adaptation condition* (MINUS EXTREME, ORIGINAL, PLUS EXTREME) was conducted. With the given test power, we were able to detect a large main effect for adaptation condition, *F*(2,33) = 6.01; *p* = 0.006; ${\upeta }_{\mathrm{p}}^{2}$ = 0.267. Pairwise comparisons showed a significant difference between the MINUS EXTREME (*M* = -1.96; *SD* = 0.11) and PLUS EXTREME (*M* = 0.43; *SD* = 0.19) adaptation condition, *t*(33) = -3.05; *p* = 0.013. Therefore, faces with slightly increased complexion were significantly chosen more often as the original face after adaptation to PLUS EXTREME faces than after adaptation to MINUS EXTREME faces, where participants chose the slightly decreased version as the original face. There was also a significant difference between MINUS EXTREME and ORIGINAL (*M* = 0.35; *SD* = 0.26; *t*(33) = − 2.95, *p* = 0.017; Bonferroni corrected): faces with slightly decreased complexion were chosen more often in the MINUS EXTREME adaptation group as the original face in comparison to the ORIGINAL adaptation group. There was no difference between ORIGINAL and PLUS EXTREME (*t*(33) = -0.10, *p* = 1.00; Bonferroni corrected). There was no effect for transfer level, *F*(2,66) = 0.62; *p* = 0.940; ${\upeta }_{\mathrm{p}}^{2}$ = 0.002, and both factors did not interact, *F*(4,66) = 1.13; *p* = 0.351; ${\upeta }_{\mathrm{p}}^{2}$ = 0.064. The overall pattern of results can be retrieved from Fig. 5.

## Discussion

The aim of the present study was to increase the knowledge about adaptation effects to very natural and non-configural changes in faces (based on [21]). A very natural “manipulation” of the appearance of faces through exposure to sunshine is a change in complexion. Pre-experimentally familiar faces of well-known celebrities were used. One could argue that brightness manipulations done by Mueller et al. [21] would be very similar to the changes in complexion level done by the present study. It is therefore important to note that – as described in the methods section – participants actually could clearly identify that the used stimuli faces were changed regarding their brightness [21] or their complexion (the present study). Therefore, the present data can be interpreted as the result of a new manipulation of non-configural face information.

Mesik et al. [9] showed that adaptation seems to result from a combination of multiple temporally-tuned processes (short-term and long-term in nature). The more long-lasting processes seem to refer to a (visual) memory component. Since the robustness and sustainability of face adaptation effects were found to be remarkable, the present study also focused on this memory component of facial adaptation aftereffects and not on a mere perceptual (short-term) component of these effects. By using a paradigm separating adaptation and test phase by approximately five minutes (see [14]), we could probably test if those adaptation effects are more representationally (and therefore memory-) related. In our results, we observed clear adaptation effects (with a large effect size demonstrating a clear difference between MINUS and PLUS EXTREME adaptation groups); this is particularly interesting as we were able to show a robustness of these effects even after a 5-min break.

Our study, therefore, seems to actually investigate the more sustainable adaptation mechanism of the different mechanisms proposed by Mesik et al. [9] and Bao, Fast, Mesik, and Engel [27]. The effect seems to refer to a representationally (memory-) related component—although this might not be undisputed as the definition of when perception-effects end and memory-effects start is arbitrary. So any proposal of a memory-based effect is based on a more liberal definition of what a memory effect is—only further research, for instance, by analyzing the distribution of brain activities across typical memory-related areas such as the temporal lobe, can shed more light on this critical debate on the origin of such adaptation effects. Complementary to the adaptation effects by Mueller et al. [21], who show adaptation effects with artificial brightness manipulations, we could reveal similar effects to natural changes in faces we are confronted with in everyday life. We cannot completely rule out that other theoretical accounts, such as priming or retinal effects, can explain our data. So, only future research efforts might provide more critical tests for our hypothesis of a representational basis of the effects. But at least by incorporating previous studies using the same or very similar paradigms (e.g., [12]) in our overall analysis, we are able to document strong and robust adaptation effects which last at least one week and which can be qualified as effects beyond episodic memory (see [17]). It is true that our study did show adaptation effects only lasting from about five minutes up to about 30 min, however it points in the direction where we cannot exclude more long-lasting effects completely. Another indication for a memory-related interpretation of the effects (and therefore against a retinal effect) is the procedure we employ, where faces in adaptation and test phase are shown at different places. No pure short-living retinal or priming effects could explain that adaptation takes place. Furthermore, that adaptation takes place even on the structural level, i.e., not only concerning the same depiction of one person. Ditye et al. [28] showed additionally that sleep facilitates face adaptation, another hint that facial adaptation might be a memory-related effect. Finally, our results align with Schneider and Carbon’s [13] recent EPM explaining facial representations in memory. The EPM argues for a fast memory update through efficient storage mechanisms (e.g., giving more flexibility with dynamic changes in faces such as tanning).

Since there was no difference between ORIGINAL and PLUS EXTREME, the effect seems to be shifted in the direction of MINUS EXTREME. A very strong adaptation effect of the MINUS EXTREME adaptation group and/or by a relatively weaker adaptation effect of the PLUS EXTREME adaptation group, and/or a shift of the control group toward the minus pole, could be the reason for this shift. Thus, after being exposed to an adaptor face strongly lightened in complexion, participants also tend to select images lightened in complexion in the test phase when being asked to choose the veridical image out of the presented images. Compatible with the rationale provided by Mueller et al. [21], one reason for this selective adaptation could be that an increase in complexion intensity is more obvious as image manipulation. Hence, participants would identify images with higher complexion intensity as manipulated images more easily and would reject them when asked to choose the veridical. The difference between the non-manipulated original image and the image with slightly decreased complexion intensity may be harder to identify so participants would select the image with lighter/ lower complexion in some trials. Also, there could be an interference of face processing in the present study with the so-called “other-race” effect (showing that people are generally better at recognizing or identifying faces of their own ethnicity, compared to faces of other ethnicities). We aimed at simulating stronger tanning and not at changing the faces in the direction of another ethnicity. Such recognition as another ethnicity is known to lead to qualitatively different face processing, for instance, amplifying stereotypical features, as intriguingly shown by MacLin and Malpass [29]. This other-race effect is well documented for unfamiliar faces. As we employed familiar faces in our experiment, we do not think that participants flexibly change the assignment of a well-known face to another ethnical group. Additionally, we also believe that participants did not misperceive the manipulation of complexion as an ambient factor, given the additional study dataset, briefly mentioned above (see Fig. 3). On the contrary, participants seem to have identified our graphical manipulation as an inherent facial property, as documented in this dataset.

However, the shift of the adaptation effect towards the negative pole could be simply a sign of different adaptivity to individual changes and the result of natural fluctuations: the adaptor was manipulated on a mere physical way, which does not tell us how exactly the impact on the subjective experience level was. Seemingly, the lack of the effect in the PLUS EXTREME condition indicated that the adaptation effect was less effective for the PLUS EXTREME manipulations. One could also argue that it is an effect explained by a general response bias, i.e., a general tendency to respond “incorrectly” (not choosing the veridical face). The expected response pattern would then most likely be random, i.e., choosing the lighter or darker version equally likely. Our data, however, reveal not such a random pattern, but a clear shift towards the adaptor (MINUS EXTREME) which is compatible with a rather systematic adaptation process. Also, this lack of effect towards the PLUS EXTREME condition could be due to more subtle shifts in mental representations, i.e., smaller than + 25%, that could not be detected with the design used in the present study. Future studies should investigate this possibility by using a response format with more alternatives to choose from, i.e., faces with different and finer-graded complexion intensities. Also, they should investigate possible anomalies in the perception of different complexion intensities (and possible interference with the other-race effect) to clarify the underlying factors for the bias identified in the present study and by Mueller et al. [21].

Looking closer at the presented faces, although most celebrities employed here were White European faces, some show darker original facial complexion. It would be interesting for future studies to look closer to the possible differences in the effectiveness of the used complexion levels for different ethnicities. Additionally, perceivers’ level of experience with tanning, attitude towards tanning, and the precise level of facial complexion as experimental factors would have to be taken into consideration, which was beyond the scope of the present study.

The adaptation effects appeared across all transfer levels (pictorial, structural, cross-identity), indicating that the uncovered effects were not bound to specific pictorial depictions but generalized across different depictions and even other faces in a similar way. Therefore, we assume that our adaptation effects address higher face concepts (e.g., a generic norm of faces). This indicates that exposing people to faces of certain levels of different complexions lets people adapt to general levels of complexion independently from specific exemplars with which the participants were familiarized. Adaptation on the cross-identity level as observed in the present study, seems to represent a shift in the norm of skin tone in general. In terms of the theory of face space (see [30]), this would mean that the whole face space changes towards typical visual conditions of previously experienced facial depictions (in our case, the intensity of changes in complexion). Also, our data align with the mentioned EPM by Schneider and Carbon [13], where the authors assume that our mental representation of faces is more flexible than expected. The episodic prototypes consist of only a few needed prototypes leading to efficient storage and more flexibility with dynamic changes in faces (such as tanning). Therefore, our data could represent a change in the stored episodic prototypes of the presented celebrities.

Other studies addressing configural face adaptation effects found weaker adaptation effects on the structural level compared to the pictorial level (e.g., [17, 31]) and also weaker effects for cross-identity level in comparison to both other levels (e.g., [12, 14, 16]; see also review [18]). This could be partially due to the stimuli used in the present study, representing highly natural changes in faces. Since studies reported above with similar experimental designs could show differences concerning the different transfer conditions, it seems that in the present study with the used stimulus material, we can show that adaptation to changes in complexion is actually not an image-specific effect but an adaptation to the current conditions/context.

To conclude, complexion intensity information seems to be stored in memory and thus be involved in face recognition. The perception of changes in complexion seems to be very adaptive, which might be why we do not even recognize them in most situations. This seems to be surprising since a darker complexion is typically propagated in Western society, and indeed, a lot of effort is put into reaching the goal of becoming tanned. Within a very short period, adaptation processes seem to be taking place, and … no one will perceive the change anymore. Since adaptation is a major mechanism of optimizing our processing of environmental changes, it completely makes sense to adapt to changes related to tanning fast. Through adaptation, irrelevant information can be filtered out, and more resources can be used for processing more relevant information in our current environment.

## Data Availability

The dataset generated and analyzed during the current study are available in the OSF repository: https://osf.io/qe9fy/?view_only=2d200bd6fa3a48e7965ce16041a7f1b8

## Footnotes

1 The number of participants of this pre-study was not calculated a priori, but we aimed at covering different perspectives in this respect to be sure that the finally selected celebrities were probably familiar to most of the participants; furthermore the number was approximately the same as in [21, 22]).

2 Musicians (Adele, Amy Winehouse, Ed Sheeran, Justin Bieber, Justin Timberlake, Katy Perry, Pink, Rihanna, Selena Gomez), actors (Cameron Diaz, Daniel Radcliffe, Elyas M’Barek, Emma Watson, George Clooney, Jennifer Aniston, Leonardo DiCaprio, Til Schweiger, Will Smith, Brad Pitt), politicians (Angela Merkel, Barack Obama, Donald Trump, Queen Elizabeth, Vladimir Putin), athletes (Boris Becker, Cristiano Ronaldo, Joachim Löw), models (Heidi Klum, Paris Hilton) and entertainers (Thomas Gottschalk, Dieter Bohlen).

3 The width of a picture took about 10.9% of the monitor ‘s width (~ 55 cm), so 6.02 cm. This yields a visual angle of 5.3°.
